# Status-Based Asymmetries in Relative Deprivation During the COVID-19 Pandemic

**DOI:** 10.1177/19485506231163016

**Published:** 2023-04-04

**Authors:** Kieren J. Lilly, Chris G. Sibley, Danny Osborne

**Affiliations:** 1 University of Auckland, New Zealand

**Keywords:** relative deprivation, COVID-19 pandemic, ethnic group membership, collective action, income redistribution

## Abstract

The COVID-19 pandemic has amplified existing inequalities by disproportionately affecting marginalized groups, which should differentially affect perceptions of, and responses to, inequality. Accordingly, the present study examines the effects of the pandemic on feelings of individual- and group-based relative deprivation (IRD and GRD, respectively), as well as whether these effects differ by ethnicity. By comparing matched samples of participants assessed before and during the first 6 months of the pandemic (*N*_total_ = 21,131), our results demonstrate the unique impacts of the pandemic on IRD and GRD among ethnic minorities and majorities. Moreover, our results reveal the status-based *indirect* effects of the pandemic on support for both collective action and income redistribution via IRD and GRD. As the pandemic rages on, these results foreshadow long-term, status-specific consequences for political mobilization and support for social change.

On December 31, 2019, the first cases of the novel coronavirus (COVID-19) were reported in Wuhan, China (World Health Organization, 2020). Since then, there have been over 655 million confirmed cases of the virus across 228 countries and territories, including over 6 million deaths (as of December 15, Worldometers, 2022). In response to the deadly virus, many countries adopted “lockdown” strategies, with varying degrees of success (Bremmer, 2021; Ritchie et al., 2022). These lockdowns typically mandated staying home, closing non-essential businesses, and social distancing. As such, COVID-19 impacted all aspects of daily life (see Robinson et al., 2021), including physical and psychological health (Gavin et al., 2020; Shanbehzadeh et al., 2021), as well as socioeconomic outcomes (Brodeur et al., 2021). While these findings corroborate the impact of past epidemics, COVID-19 has far *greater* potential consequences on income distribution than its historical counterparts due to its unprecedented scale (e.g., Furceri et al., 2022).

Despite its ubiquity, the pandemic has not been felt equally. In particular, ethnic minorities are disproportionally impacted by infections, hospitalizations, and fatalities associated with COVID-19 (Mackey et al., 2021; Mathur et al., 2021). Ethnic minorities—as well as migrants and women—have also been disproportionally affected by unemployment and financial hardship during the pandemic (Dang & Viet Nguyen, 2021; Gemelas et al., 2022; Hu, 2020). Thus, while the effects of COVID-19 on the general population are undeniable, the pandemic has intensified existing inequalities and left some more disadvantaged than others.

Critically, objective deprivation only partially explains people’s feelings of dissatisfaction and injustice arising from inequality (see Schmalor & Heine, 2022; Smith et al., 2012). For example, *subjective* inequality measures better predict well-being than do objective measures (Vezzoli et al., 2022). Moreover, objectively disadvantaged groups that overlook their deprivation (relative to other groups) are less supportive of collective action to redress inequities (Osborne, García-Sánchez, & Sibley, 2019). Hence, examining the pandemic’s impact on feelings of *relative* deprivation is integral to understanding the consequences of the pandemic on social change and efforts to redress inequality (see Grant & Smith, 2021).

To these ends, the present study assesses the impact of the pandemic on individual- and group-based relative deprivation (IRD and GRD, respectively). Specifically, we draw on a large, nationwide sample of participants collected in the first 6 months of the pandemic (i.e., March–August 2020) and compare them with a propensity-matched control sample who completed the survey in 2019, well before the first reported cases of COVID-19. In addition, given that New Zealand’s Alert Level system began with a wide-scale set of restrictions that gradually eased during these first 6 months (New Zealand Government, 2021; see Table 1), we compare participants’ responses in different levels of “lockdown.” Critically, we examine (a) whether the effects of the pandemic differ between ethnic minorities and majorities and (b) whether the different lockdown conditions are *indirectly* associated with support for ethnic-based collective action and income redistribution via IRD and GRD.

**Table 1:** COVID-19 Timeline and Demographic Information for Participants Included in Our Analyses

Covid-19 timeline	Pre-pandemic	Pandemic (total)	Lockdown Alert Level 4	Alert Level 3	Alert Level 2	Alert Level 1	Auckland Alert Level 3
	Propensity-matched control	March 26, 2020−August 30, 2020	March 26, 2020−April 27, 2020	April 28, 2020−May 13, 2020	May 14, 2020−June 08, 2020	June 09, 2020−August 11, 2020	August 12, 2020−August 30, 2020
Sample size	10,667	10,464	2,952	1,281	1,516	3,422	1,293
**Gender** (women)	64.7% (6899)	65.0% (6799)	65.4% (1931)	67.5% (865)	69.1% (1048)	61.2% (2094)	66.6% (861)
Age, *M* (*SD*)	53.05 (13.26)	53.63 (14.06)	54.01 (13.85)	53.65 (15.90)	53.45 (14.69)	53.39 (13.32)	53.61 (13.77)
**Birth year, *M*** (*SD*)	1966 (13.26)	1966 (14.08)	1966 (13.86)	1966 (15.90)	1966 (14.69)	1967 (13.34)	1967 (13.78)
**Ethnicity** (yes)							
European/Pakeha	84.7% (9,031)	83.4% (8,732)	84.9% (2,505)	84.5% (1,083)	86.5% (1,312)	80.2% (2,743)	84.2% (1,089)
Māori	10.4% (1,109)	11.3% (1,184)	10.9% (322)	10.9% (140)	9.0% (137)	13.1% (448)	10.6% (137)
Pacific	1.7% (181)	1.9% (181)	1.6% (46)	1.3% (17)	2.4% (36)	2.1% (72)	2.0% (26)
Asian	3.2% (346)	3.4% (351)	2.7% (79)	3.2% (41)	2.0% (31)	4.6% (159)	3.2% (41)
**Born in NZ** (yes)	76.6% (8,176)	76.5% (8,010)	76.4% (2,256)	75.5% (967)	73.9% (1,121)	78.7% (2,692)	75.3% (974)
**NZ citizen** (yes)	92.8% (9,902)	92.7% (9,701)	93.0% (2,745)	91.8% (1,176)	91.5% (1,387)	93.4% (3,195)	92.7% (1,198)
**Education**,^a^*M* (*SD*)	5.79 (2.65)	5.78 (2.67)	5.91 (2.64)	5.96 (2.67)	6.11 (2.62)	5.39 (2.70)	5.94 (2.58)
Employed (yes)	75.9% (8,092)	73.0% (7,636)	72.4% (2,136)	69.7% (893)	71.6% (1,086)	75.0 (2,566)	73.9% (955)
Income,^b^*M* (*SD*)	1.18 (1.44)	1.14 (1.11)	1.12 (1.21)	1.07 (1.13)	1.18 (1.31)	1.15 (0.97)	1.16 (0.90)
**NZDep**,^c^*M* (*SD*)	4.76 (2.68)	4.87 (2.67)	4.98 (2.71)	4.96 (2.64)	4.85 (2.69)	4.80 (2.64)	4.74 (2.67)
**NZSEI**,^d^*M* (*SD*)	56.33 (15.84)	56.19 (15.75)	56.61 (15.84)	56.74 (15.79)	57.58 (15.49)	54.75 (15.79)	57.00 (15.45)
**Urban** (yes)	82.0% (8,749)	81.2% (8,498)	82.3% (2,430)	83.9% (1,075)	81.8% (1,240)	79.4% (2,716)	80.2% (1,037)
Auckland (yes)	24.7% (2,631)	25.7% (2,689)	24.8% (731)	24.4% (313)	25.3% (383)	26.2% (897)	28.2% (365)
**Religious** (yes)	33.9% (3,612)	33.7% (3,526)	33.4% (986)	33.0% (423)	32.1% (487)	34.5% (1182)	34.6% (448)
**Partner** (yes)	72.3% (7,709)	71.5% (7,482)	70.7% (2,087)	65.3% (837)	68.0% (1,031)	74.6% (2,552)	75.4% (975)
**Parent** (yes)	74.4% (7,936)	74.6% (7,804)	73.9% (2,182)	67.8% (868)	72.2% (1,094)	77.5% (2,652)	78% (1,008)
**Smoker** (yes)	6.5% (697)	7.0% (729)	6.8% (202)	8.9% (114)	7.5% (114)	6.7% (228)	5.5% (71)
**Disability** (yes)	28.6% (3,046)	28.6% (2,996)	30.6% (902)	32.9% (422)	33.6% (510)	23.3% (798)	28.2% (364)
**Diagnosis** (yes)							
Depression	17.0% (1,811)	17.0% (1,776)	17.7% (522)	20% (256)	19.9% (301)	14.0% (480)	16.8% (217)
Anxiety	12.8% (1,369)	12.5% (1,307)	12.6% (371)	14.2% (182)	15.4% (233)	10.6% (362)	12.3% (159)

*Note*. Variables highlighted in bold indicate propensity score matching variables used to match participants in the pandemic condition to the pre-pandemic control sample.

a Education (0 = no formal qualification, 10 = doctoral degree). ^b^ Divided by US$100,000. ^c^ New Zealand Deprivation Index. ^d^ New Zealand Socioeconomic Index.

## Relative Deprivation Theory

Beginning with Stouffer et al.’s (1949) war-time studies, relative deprivation theory argues that responses to inequality depend on a person’s *subjective* comparisons with similar others rather than their objective (dis)advantage (Pettigrew, 2015; Smith & Huo, 2014). Runciman (1966) expanded this concept by distinguishing between egoistic (individual) and fraternal (group) relative deprivation—an individual can believe they are deprived relative to other individuals (IRD) or that their ingroup is deprived relative to other groups (GRD). These discrete comparison targets produce different “yardsticks” to which individuals measure their (or their ingroup’s) position in society (Kim et al., 2018; Smith & Huo, 2014).

Consistent with Runciman’s (1966) distinction, IRD best predicts individual-based outcomes, including reduced well-being and mental health (Osborne et al., 2012; Osborne, Sibley, & Sengupta, 2015; Smith et al., 2020; Walker, 1999). Conversely, GRD best predicts group-based outcomes, including intergroup bias (Guimond & Dambrun, 2002; Pettigrew et al., 2008) and collective action support (Abrams & Grant, 2012; Mummendey et al., 1999). Critically, these constructs produce larger effects than do objective deprivation measures (see Smith et al., 2012). Thus, one’s subjective *interpretation* of their position in society is paramount for understanding their responses to objective inequality.

Although measuring the effects of COVID-19 on objective indicators is important, it is critical to assess the pandemic’s impact on one’s *relative* position. Indeed, the pandemic presents unique challenges that should elicit IRD or GRD, depending on the type of comparison (see Osborne, Sibley, & Sengupta, 2015). For example, the unprecedented financial hardship associated with COVID-19 (e.g., Cortes & Forsythe, 2022) collapsed the global economy, with job loss up to four times higher than the 2009 global financial crisis (United Nations, 2021). However, economic hardship arose in tandem with stark increases in *wealth* for the elite (Neate, 2020; Ryan, 2022). Such inequities should emphasize *individual* economic conditions and foster social comparisons (see Cheung & Lucas, 2016). The salience of income inequality during the pandemic should thus increase IRD among the general population, particularly during the strictest lockdown conditions where these effects were most pronounced (Fletcher et al., 2022; Prickett et al., 2020).

The pandemic also emphasized *group*-based inequalities and, as such, should increase GRD. Although ethnic minorities were disproportionally affected by unemployment and economic hardship *before* the pandemic (see Iceland, 2019; Pager & Shepherd, 2008), the pandemic exacerbated these trends by differentially impacting job and income loss (Gemelas et al., 2022; Hu, 2020; Katikireddi et al., 2021). The salience of these inequities should thus increase GRD among ethnic minorities.

Recent research supports these theses and demonstrates the unique effects of the pandemic on people’s perceptions of, and attitudes toward, inequality. For example, the pandemic altered people’s attributions for poverty (Wiwad et al., 2021), elicited frustration over class-based inequalities (Ravenelle et al., 2022), and increased inequality *aversion* (Asaria et al., 2021). Moreover, Kiebler and Stewart (2021) show that IRD increased among low-income students during the pandemic. Thus, the pandemic (re)shaped people’s frustration and attitudes toward injustice, creating a unique context to study feelings of relative deprivation (see Grant & Smith, 2021).

Indirect support for our hypotheses also comes from research revealing that distinct forms of objective inequality elicit individual- or group-based comparisons. For example, Osborne, Sibley, and Sengupta (2015) demonstrate that personal income correlates negatively with IRD, while neighborhood-level deprivation correlates positively with GRD. Similarly, the objective disadvantages faced by people of low subjective socioeconomic status (SES) elicit feelings of personal deprivation (Greitemeyer & Sagioglou, 2016, 2017). That is, individual- and group-based objective circumstances promote greater individual- and group-based *relative* comparisons, respectively. The pandemic’s unique effects on individual- and group-level circumstances should thus elicit IRD and GRD, respectively.

## Study Overview

The current study examines the impact of the pandemic on feelings of IRD and GRD. To do so, we compare propensity-matched samples of New Zealanders who completed our survey *before* the pandemic (October 01–December 31, 2019) to those who completed the survey during the first 6 months of the pandemic (March 26–August 30, 2020). Propensity-score matching strengthens causal inferences by providing a matched “control” sample when random assignment to a treatment group is impossible (Austin, 2011). There are, however, limitations to propensity-score matching, as unmatched variables may account for group differences that would be controlled for in experiments via randomization. To minimize this limitation, we match participants on objective deprivation (e.g., SES), demographic covariates (e.g., gender, age, and ethnicity), and other socioeconomic and health indicators (for a complete list, see Table S1). We thus increase confidence that our analyses uniquely examine the impact of the pandemic on participants’ IRD and GRD (relative to the matched control sample).

We investigate these effects in the context of New Zealand’s four-tier Alert Level System for the COVID-19 pandemic (New Zealand Government, 2021; see Table 1). On March 25, 2020, New Zealand entered a national lockdown (Alert Level 4), which required people to stay at home save for essential movement. On April 27, 2020, New Zealand entered Alert Level 3, which eased restrictions slightly by allowing 10-person gatherings for weddings and funerals. On May 13, 2020, Alert Level 2 allowed businesses to reopen (with social distancing) and permitted gatherings of up to 100 people. Alert Level 1 began on June 08, 2020, which eased restrictions back to “normal” with no social distancing or limits on social gatherings. However, on August 12, 2020, Auckland (New Zealand’s largest city) returned to Alert Level 3 following a second community outbreak.^1^ Because these Alert Levels capture restrictions of increasing severity, we investigate the potentially different effects of the Alert Levels on our focal variables.

Although the pandemic increased the salience of *objective* personal- and group-based inequalities, the unique challenges of each Alert Level should have differential impacts on relative deprivation. Given the unique financial pressures and salience of income inequality during the pandemic, participants should report greater feelings of IRD during the pandemic relative to those in the pre-lockdown control group. In addition, Alert Levels with the strongest restrictions presented unprecedented hardships and, thus, should elicit larger increases in IRD than less restrictive Alert Levels.

Likewise, GRD should increase during the pandemic relative to the control group. However, minority groups are overrepresented in COVID-19 unemployment statistics and experience disproportionate rates of COVID-19-related infections, hospitalizations, and deaths (Gemelas et al., 2022; Hu, 2020; Ministry of Health, 2022). Because the pandemic exacerbated existing ethnic inequalities, GRD should be particularly heightened among ethnic minorities.

We also examine the *indirect* effects of the different Alert Levels on support for (a) ethnic-based collective action and (b) income redistribution via IRD and GRD. Because perceiving injustice—particularly when one is *angry* or *frustrated* by their ingroup’s status (Jost et al., 2017; Smith et al., 2012; Thomas et al., 2020)—is a necessary antecedent to collective action, the pandemic should shape support for these social issues *through* relative deprivation (Grant & Smith, 2021). By examining this thesis, we contribute to a growing literature examining the effects of the pandemic on subjective experiences of, and responses to, inequality.

## Method

### Sampling Procedure and Participants

We analyzed data from Time 11 of the New Zealand Attitudes and Values Study (NZAVS)—an ongoing nationwide longitudinal panel study of New Zealand adults that began in 2009. Participants were initially sampled from the New Zealand electoral roll and represent New Zealand’s general population in age, SES, and region of residence (see Sibley, 2021). We focus on time 11 (*N* = 42,684), as data collection occurred between October 2019 and September 2020, including the first 6 months of the pandemic (March–August 2020). Because data collection began before the pandemic, a priori power analyses were not conducted.

A total of 21,131 participants provided partial or complete responses to our variables of interest (*M_age_* = 53.34, *SD* = 13.66). Participants were predominantly women (64.8%) and employed (74.8%), and a quarter lived in Auckland (25.2%). Participants identified as New Zealand European (84.1%), Māori (10.9%), Asian (3.3%), and Pasifika (1.8%). Table 1 displays demographic information by condition.

We used propensity-score matching to match the respondents who completed the survey during the pandemic (*N* = 10,464) with respondents from a pool of pre-pandemic “controls” (*N* = 10,667) on a range of socioeconomic and demographic factors (see Table S1). Participants in the control group completed the questionnaire between October 2019 and December 2019, before the first cases of COVID-19 were reported (see Sibley et al., 2020).

### Measures

Unless noted, items were measured on a 1 (*strongly disagree*) to 7 (*strongly agree*) scale and averaged to assess their respective constructs.

#### Predictors

##### Alert Levels

Participants in the pandemic condition completed the questionnaire within New Zealand’s Alert Level System (New Zealand Government, 2021; see Table 1). The different Alert Level conditions and the matched control sample were dummy-coded (0 = no, 1 = yes) so that the effects reflect differences between the pre-pandemic control and the given Alert Level.

##### Minority Status

Minority status was dummy-coded (0 = New Zealand European, 1 = Minority).

#### Outcome Variables

**IRD** was assessed using two items adapted from Abrams and Grant (2012): (a) “I’m frustrated by what I earn relative to other people in New Zealand” and (b) “I generally earn less than other people in New Zealand” (α = .59).

**GRD** was assessed using two items adapted from Abrams and Grant (2012): (a) “I’m frustrated with what my ethnic group earns relative to other groups in New Zealand” and (b) “People from my ethnic group generally earn less than other groups in New Zealand” (α = .62).

**Collective action support** was measured using three items from the work of Cronin et al. (2012): (a) “I have considered voting in terms of what is good for my particular ethnic group”; (b) “I have considered participating in demonstrations on behalf of my ethnic group”; and (c) “I have considered signing petitions on behalf of my ethnic group” (α = .78).

**Support for income redistribution** was measured by asking participants how strongly they oppose or support “Redistributing money and wealth more evenly among a larger percentage of the people in New Zealand through heavy taxes on the rich.”

### Covariates

Gender (0 = woman, 1 = man), age, income, employment status (0 = no, 1 = yes), and education were used as demographic covariates. Education was coded on a 0 (no formal qualification) to 10 (doctoral degree or equivalent) scale.

## Results

We regressed IRD and GRD simultaneously onto our predictors in two separate models. In the first model, we regressed IRD and GRD onto the different Alert Levels, minority status and our covariates. The second model included interaction terms for each Alert Level with minority status.^2^Table 2 displays the descriptive statistics and bivariate correlations for the variables included in this study. The complete questionnaire and syntax used in this study are available at: https://osf.io/nzev8/.

**Table 2:** Descriptive Statistics and Bivariate Correlations Between the Variables Included in our Analyses

Variable	1	2	3	4	5	6	7	8	9	10	11	12	13
01. Gender^a^	—												
02. Age	.083***	—											
03. Minority^b^	−.020**	−.107***	—										
04. Education	−.061***	−.130***	−.027***	—									
05. Employed^c^	.022***	−.314***	.015*	.137***	—								
06. Income^d^	.057***	−.105***	−.022***	.154***	.199***	—							
07. Alert Level 4^c^	−.005	.020**	−.009	.019**	−.021**	−.012	—						
08. Alert Level 3^c^	−.014*	.006	−.003	.017*	−.029***	−.018*	−.102***	—					
09. Alert Level 2^c^	−.025***	.002	−.019**	.034***	−.019**	.004**	−.112***	−.071***	—				
10. Alert Level 1^c^	.033***	.002	.047***	−.066***	.008	−.002	−.177***	−.112***	−.122***	—			
11. Alert Level 3^e^	−.009	.005	−.001	.015*	−.004	.000	−.103***	−.065***	−.071***	−.112***	—		
12. IRD	−.085***	−.074***	.062***	−.156***	−.132***	−.262***	.013	.020**	.016*	−.008	.004	—	
13. GRD	−.037***	−.026***	.485***	−.092***	−.030***	−.067***	−.001	.002	−.009	.047***	.011	.234***	—
$\bar{x}$	0.35	53.34	0.16	5.79	0.75	1.16	0.14	0.06	0.07	0.16	0.06	3.36	2.27
*SD*	0.478	13.66	0.37	2.66	0.43	1.29	0.35	0.24	0.26	0.37	0.24	1.55	1.40
α	—	—	—	—	—	—	—	—	—	—	—	.588	.617
*N*	21,131	21,131	21,131	21,131	21,036	21,131	21,131	21,131	21,131	21,131	21,131	20,834	21,113

*Note.* IRD = individual-based relative deprivation; GRD = group-based relative deprivation.

a Dummy-coded (0 = women, 1 = men). ^b^ Dummy-coded (0 = New Zealand European, 1 = Minority). ^c^ Dummy-coded (0 = no, 1 = yes). ^d^ Divided by US$100,000. ^e^ Alert Level 3 in Auckland only, Alert Level 2 elsewhere, dummy-coded (0 = no, 1 = yes).

* *p* < .05. **p < .01. ***p < .001.

In terms of IRD, Model 1 reveals that women (*b* = −0.198, *SE* = 0.021, *p* < .001) and the unemployed (*b* = −0.377, *SE* = 0.009, *p* < .001) scored higher on IRD than did men and employed participants, respectively (see Table 3). In addition, age (*b* = −0.016, *SE* = 0.001, *p* < .001), education (*b* = −0.073, *SE* = 0.004, *p* < .001), and income (*b* = −0.316, *SE* = 0.005, *p* < .001) correlated negatively with IRD. Conversely, minority group status correlated positively with IRD (*b* = 0.162, *SE* = 0.028, *p* < .001).

**Table 3:** Multiple Regression Analyses Predicting Individual-Based (IRD) and Group-Based Relative Deprivation (GRD) During the COVID-19 Pandemic

	Individual-based relative deprivation (IRD)						Group-based relative deprivation (GRD)					
	Model 1			Model 2			Model 1			Model 2		
	*B*	*SE*	β	*B*	*SE*	β	*B*	*SE*	β	*B*	*SE*	β
Intercept	5.300***	0.058	3.427***	5.295***	0.058	3.424***	2.238***	0.049	1.599***	2.252***	0.049	1.609***
Gender	−0.198***	0.021	−0.061***	−0.197***	0.021	−0.061***	−0.085***	0.018	−0.029***	−0.085***	0.018	−0.029***
Age	−0.016***	0.001	−0.143***	−0.016***	0.001	−0.143***	0.001	0.001	0.008	0.001	0.001	0.009
Minority	0.162***	0.028	0.038***	0.201***	0.040	0.047***	1.840***	0.023	0.481***	1.722***	0.033	0.450***
Education	−0.073***	0.004	−0.125***	−0.073***	0.004	−0.125***	−0.036***	0.003	−0.069***	−0.036***	0.003	−0.069***
Employed	−0.377***	0.009	−0.106***	−0.377***	0.025	−0.106***	−0.050*	0.021	−0.015*	−0.049*	0.021	−0.015*
Income	−0.316***	0.025	−0.247***	−0.316***	0.009	−0.247***	−0.052***	0.007	−0.045***	−0.053***	0.007	−0.046***
Alert Level 4 (AL4)	0.076*	0.030	0.017*	0.108**	0.033	0.024***	0.047	0.025	0.012	0.017	0.028	0.004
Alert Level 3 (AL3)	0.112**	0.043	0.017**	0.115*	0.047	0.018*	0.052	0.036	0.009	0.018	0.039	0.003
Alert Level 2 (AL2)	0.133***	0.040	0.022***	0.160***	0.043	0.027***	0.042	0.033	0.008	0.003	0.036	0.000
Alert Level 1 (AL1)	−0.028	0.029	−0.007	−0.031	0.032	−0.007	0.101***	0.024	0.027***	0.067**	0.027	0.018*
Alert Level 31 (AL3)	0.062	0.043	0.010	0.054	0.047	0.008	0.101**	0.036	0.017**	0.041	0.039	0.007
Interactions												
AL4 × Minority				−0.215*	0.084	−0.020*				0.194**	0.071	0.020**
AL3 × Minority				−0.021	0.119	−0.001				0.224*	0.100	0.015*
AL2 × Minority				−0.195	0.116	−0.012				0.278**	0.098	0.019**
AL1 × Minority				0.009	0.074	0.001				0.198***	0.062	0.025***
AL3^a^× Minority				0.051	0.119	0.003				0.388***	0.099	0.027***
Model summary												
*R*^2^	0.122***			0.122***			0.245***			0.246***		

a Alert Level 3 in Auckland only, Alert Level 2 elsewhere.

* *p* < .05. **p < .01. ***p < .001.

After adjusting for these associations, participants in Alert Level 4 (*b* = 0.076, *SE* = 0.030, *p* = .013), Alert Level 3 (*b* = 0.112, *SE* = 0.043, *p* = .009), and Alert Level 2 (*b* = 0.133, *SE* = 0.040, *p* = .001) were higher in IRD than the control group. In contrast, Alert Level 1 and the Auckland Level 3 lockdown did not differ from the propensity-matched control group on IRD (*b* = −0.028, *SE* = 0.029, *p* = .337; *b* = 0.062, *SE* = 0.043, *p* = .150). Interestingly, the effects of Alert Level 2 were stronger than that of the stricter Alert Levels, suggesting that IRD increased in the later, less restrictive, lockdown period.

As shown in Model 2, minority status only moderated the relationship between Alert Level 4 and IRD (*b* = −0.215, *SE* = 0.084, *p* = .011). The remaining interaction effects were non-significant^3^ (see Table 3), suggesting that ethnic-group differences in IRD did not vary across the remaining Alert Levels. Interestingly, simple slopes analyses revealed that the association between Alert Level 4 and IRD was positive among ethnic majorities (*b* = 0.108, *SE* = 0.033, *p* = .001), but negative and non-significant among ethnic minorities (*b* = −0.107, *SE* = 0.078, *p* = .168). Thus, only *majority* ethnic group members experienced an increase in IRD at Alert Level 4 vis-à-vis the matched control.

Turning to GRD, Model 1 reveals that women (*b* = −0.085, *SE* = 0.018, *p* < .001) and the unemployed (*b* = −0.050, *SE* = 0.021, *p* = .017) scored higher on GRD than did men and employed participants, respectively. In addition, education (*b* = −0.036, *SE* = 0.003, *p* < .001) and income (*b* = −0.052, *SE* = 0.007, *p* < .001) correlated negatively with GRD, while age was unassociated with GRD (*b* = 0.001, *SE* = 0.001, *p* = .209). As expected, minorities expressed higher levels of GRD than did ethnic majorities (*b* = 1.840, *SE* = 0.023, *p* < .001).

After adjusting for these associations, our results revealed that different Alert Levels uniquely predicted GRD (see Table 3). Specifically, only participants in Alert Level 1 (*b* = 0.101, *SE* = 0.024, *p* < .001) and Auckland Alert Level 3 (*b* = 0.101, *SE* = 0.043, *p* = .005) were higher in GRD than those in the control group. The remaining Alert Levels had comparable levels of GRD relative to the matched control group. Thus, the pandemic only increased GRD in the *latter* stages of the 2020 COVID-19 response when the lockdown effects began to accumulate.

Once again, Model 2 in Table 3 shows that minority group status moderated the associations Alert Level 4 (*b* = 0.194, *SE* = 0.071, *p* = .006), Alert Level 3 (*b* = 0.224, *SE* = 0.100, *p* = .024), Alert Level 2 (*b* = 0.278, *SE* = 0.098, *p* = .004), Alert Level 1 (*b* = 0.198, *SE* = 0.062, *p* = .001), and (Auckland) Alert Level 3 (*b* = 0.388, *SE* = 0.099, *p* < .001) had with GRD. As expected, simple slope analyses revealed that GRD increased relative to the pre-pandemic sample for ethnic minorities at Alert Level 4 (*b* = 0.211, *SE* = 0.065, *p* = .001), Alert Level 3 (*b* = 0.242, *SE* = 0.092, *p* = .008), Alert Level 2 (*b* = 0.281, *SE* = 0.091, *p* = .002), Alert Level 1 (*b* = 0.265, *SE* = 0.056, *p* < .001), and Auckland Alert Level 3 (*b* = 0.429, *SE* = 0.091, *p* < .001). Conversely, GRD among ethnic majorities did not differ from the control group across Alert Levels, except for a slight increase at Alert Level 1 (*b* = 0.067, *SE* = 0.027, *p* = .011). Thus, the pandemic uniquely increased GRD among ethnic minorities across the first 6 months of the pandemic. However, ethnic majorities began to experience an increase in GRD relative to the control group at Alert Level 1, though to a lesser degree than ethnic minorities (*b*_diff_ = 0.198, *SE* = 0.062, *p* = .001).

Given the status-specific impact of the Alert Levels on IRD and GRD, the pandemic may evoke distinct responses to inequality. Accordingly, we assessed the indirect effects of the Alert Levels on support for (a) ethnic-based collective action and (b) income redistribution via IRD and GRD. To examine whether these effects differed by ethnicity, we conducted multiple group analyses and compared models where estimates varied across groups to models where estimates were constrained to equality. Constraining the estimates to equality significantly decreased model fit for both collective action support (Δχ^2^_(17)_ = 401.85, *p* < .001) and support for income redistribution (Δχ^2^_(17)_ = 210.29, *p* < .001), suggesting that some associations differed by ethnic group membership.

**Figure 1:**
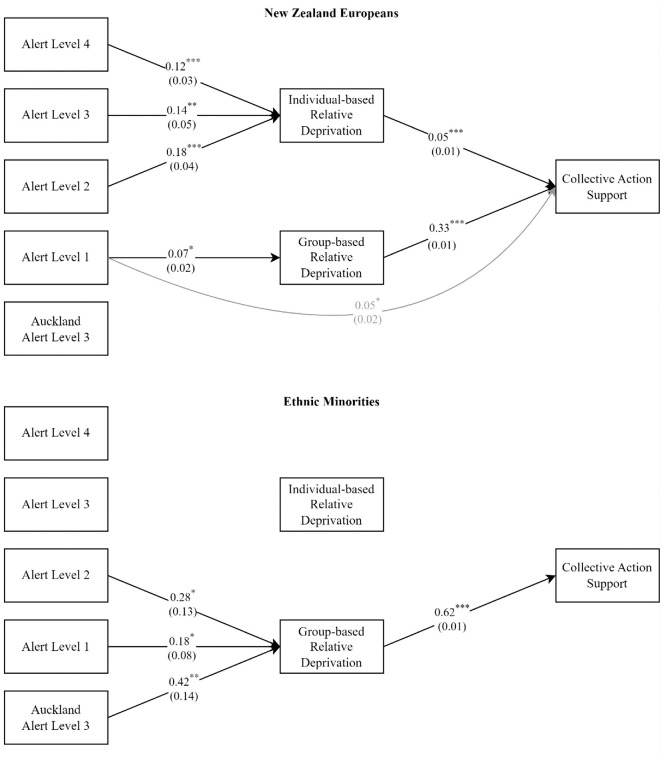
Path Analysis of the Direct and Indirect Associations Between Alert Levels, Relative Deprivation, and Collective Action Support by Ethnic Group *Note*. Estimates are unstandardized with standard errors in parentheses. Effects were estimated using 5,000 bootstrapped resamples (with replacement). For clarity, non-significant paths are excluded from the figure (see the Supplementary materials for all estimates). Paths in black display the mediation pathways. *p < .05. **p < .01. ***p < .001.

Figure 1 displays the significant associations between the Alert Levels, relative deprivation, and collective action support by ethnic group membership (for the full results, see Tables S2 and S4). Among ethnic majorities, Alert Levels 4, 3, and 2 were associated with greater collective action support via IRD, whereas Alert Level 1 was associated with greater collective action support via GRD. In contrast, IRD did not mediate associations between the pandemic and collective action support among ethnic minorities. Instead, Alert Levels 2, 1, and (Auckland) 3 were associated with greater collective action support via GRD for ethnic minorities.

**Figure 2:**
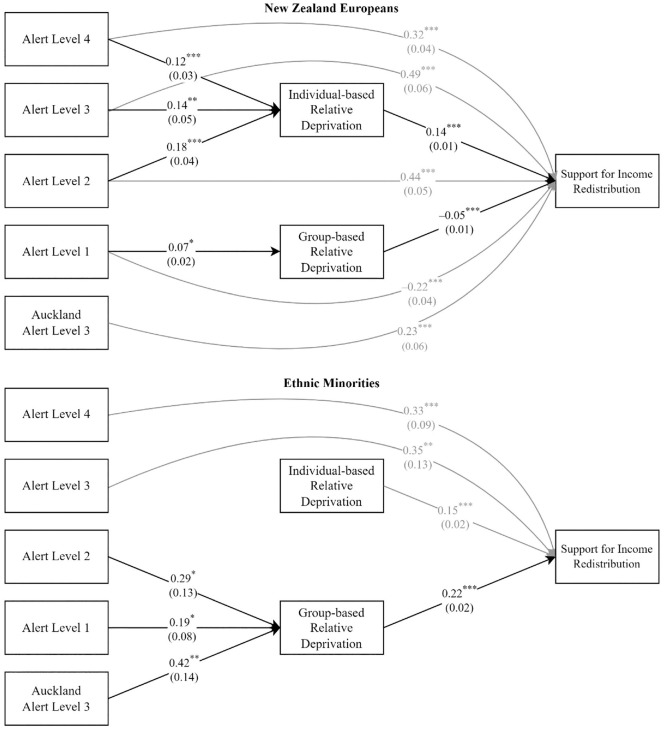
Path Analysis of the Direct and Indirect Associations Between Alert Levels, Relative Deprivation, and Support for Income Redistribution by Ethnic Group *Note*. Estimates are unstandardized with standard errors in parentheses. Effects were estimated using 5,000 bootstrapped resamples (with replacement). For clarity, non-significant paths are excluded from the figure (see the supplementary materials for all estimates). Paths in black display the mediation pathways. *p < .05. **p < .01. ***p < .001.

Regarding support for income redistribution, Figure 2 reveals that Alert Levels 4, 3, and 2 were associated with greater support for income redistribution among ethnic majorities via IRD. Interestingly, Alert Level 1 was associated with *reduced* support for income redistribution among ethnic majorities via GRD (see Tables S3 and S5). However, among ethnic minorities, IRD did not mediate any associations between the Alert Levels and income redistribution support, and Alert Levels 2, 1, and 3 (Auckland) were associated with *greater* income redistribution support via GRD. These findings suggest *status-specific* indirect associations between Alert Level 1 and support for income redistribution via GRD.

## Discussion

The COVID-19 pandemic has had unprecedented consequences for health, well-being, and the economic landscape worldwide, yet has disproportionally affected minority groups (e.g., Gemelas et al., 2022; Hu, 2020). As such, we examined the effects of the pandemic on *perceptions* of relative deprivation and whether these impacts differed across ethnic groups. Because the pandemic exacerbated existing inequalities—which should elicit greater upward social comparisons (Cheung & Lucas, 2016)—we expected IRD would increase, particularly during the strictest Alert Levels. Moreover, we expected the pandemic to increase GRD among ethnic minorities.

As hypothesized, participants in Alert Levels 4, 3, and 2 had higher levels of IRD than those in the control group. Unexpectedly, the effects of Alert Level 2 were larger than that of the stricter Alert Levels. These results may be due to the cumulative effects of the lockdown “outweighing” the initial effects of the strictest Alert Levels. Nonetheless, our results were reliable after controlling for objective deprivation indicators and other covariates, demonstrating the pandemic’s unique effect on IRD as COVID-19 began to spread, and that IRD is often rooted in reality (albeit imperfectly; see Osborne, Sibley, & Sengupta, 2015).

Interestingly, the pandemic only increased IRD during Alert Level 4 among ethnic majorities. This suggests that ethnic minorities did not feel more *personally* deprived in the initial stages of the pandemic, while ethnic majorities perceived greater IRD in the strictest “lockdown” conditions. Although unexpected, this replicates work showing that individuals are rarely high in *both* IRD and GRD (see Osborne, Sibley, Smith, & Huo, 2015). Indeed, objectively disadvantaged group members often deny being *personally* deprived but nonetheless recognize that their group as a whole is disadvantaged (see Crosby, 1984; D. M. Taylor et al., 1990). That ethnic minorities experienced higher levels of GRD—but not IRD—during the pandemic corroborates this literature. Conversely, ethnic majorities are structurally advantaged and often report greater levels of *personal* (vs group) inequities (Operario & Fiske, 2001). As such, objectively advantaged individuals may have felt less *personally* advantaged during the “strictest” lockdowns.

It is noteworthy that GRD was elevated among minorities at all Alert Levels but only during Alert Level 1 for majority group members. These results corroborate research showing that COVID-19—and subsequent lockdowns—disproportionately impacted ethnic minorities worldwide (Hu, 2020; Katikireddi et al., 2021; Mathur et al., 2021). Thus, it is unsurprising that ethnic minorities feel more collectively deprived during the pandemic.

That ethnic majorities experienced an increase in GRD during Alert Level 1 (i.e., when most restrictions had been lifted) alludes to how objectively advantaged groups may respond to the continual pressures of the pandemic. Ethnic majorities who feel collectively deprived are more likely to *oppose* efforts to redress inequality, as doing so conflicts with their self-interest (Leviston et al., 2020; Osborne, Jost, et al., 2019; Pettigrew et al., 2008; M. C. Taylor, 2002). In the pandemic context, ethnic majorities who perceive themselves as less *relatively* advantaged may be less likely to support efforts to redress inequalities. Indeed, our mediation analyses revealed status-specific indirect associations between the pandemic and support for collective action and income redistribution via IRD and GRD. Namely, the pandemic increased support for collective action via IRD (Alert Levels 4, 3, and 2) and GRD (Alert Level 1) for ethnic majorities, and only via GRD (Alert Levels 2, 1, and 3 [Auckland]) for ethnic minorities. Moreover, although the pandemic *increased* support for income redistribution among ethnic majorities via IRD (Alert Levels 4, 3, and 2) and among ethnic minorities via GRD (Alert Levels 2, 1, and 3 [Auckland]), Alert Level 1 *decreased* support for income redistribution among ethnic majorities via GRD. That is, the pandemic’s effects on GRD among ethnic majorities (a) increased collective action support on behalf of the dominant group and (b) *reduced* support for income redistribution. Given that August 2020 (Alert Level 1) marked the beginning of far-right anti-lockdown protests in New Zealand (Molyneux & Satherley, 2020; Pearse, 2020), the relationship between the pandemic and relative deprivation among majority group members may illustrate how advantaged groups respond to situational factors that increase inequality.

In addition to important practical implications, our results corroborate Runciman’s (1966) assertion that IRD and GRD develop from distinct comparison processes. Indeed, IRD and GRD increased under different Alert Levels and among different ethnic groups, highlighting that, while correlated constructs, they emerge from distinct processes and are uniquely affected by one’s environment. Moreover, our study implemented an innovative methodology which allowed us to approximate experimental conditions (Austin, 2011). While propensity-score matching may allow for unmatched variables to explain group differences, the inclusion of indicators of objective deprivation closely associated with relative deprivation (i.e., income, employment, and education) and other relevant covariates increases confidence that the pandemic uniquely impacted IRD and GRD.

Our ability to compare participants who experienced the pandemic to those who completed the study *before* the pandemic began is a novel strength of our study, as only a few studies have data to compare these conditions directly (see also Howard et al., 2022; Sibley et al., 2020). Moreover, our data spans the first 6 months of the pandemic, allowing us to compare the effects of different restriction levels. This provides a broader understanding of the effects of the pandemic on relative deprivation than studies utilizing data from only the initial lockdown(s) in March 2020. Such information is critical should another pandemic emerge requiring strict lockdowns.

Despite these strengths, our measures only assessed the *fiscal* component of relative deprivation and, as such, may not generalize to forms of relative deprivation that focus on interpersonal treatment. However, fiscal dimensions of relative deprivation are particularly relevant to the pandemic, given the economic consequences of COVID-19 on individuals and groups (Fletcher et al., 2022; Hu, 2020). In addition, our measures included cognitive and affective measures of relative deprivation, which are integral to the experiences of relative deprivation (see Smith et al., 2012). As such, we are confident that our measures accurately reflect their respective constructs and can generalize beyond our sample population.

We should also note that the current study does not reflect within-person *changes* in relative deprivation throughout the pandemic, as most of our sample had not completed the previous wave (i.e., the year before the pandemic began). Instead, we identified important differences *between* those in the pre-pandemic condition and those in the different Alert Levels. Future research should investigate whether people experienced a within-person *change* in relative deprivation before and during the pandemic.

Finally, we investigate the pandemic from March to August 2020 in a country whose initial response was highly successful but unique in its quick implementation of strict lockdowns (Cumming, 2022). As such, our results may not generalize outside the initial wave of the pandemic nor to countries with less immediate and restrictive responses to COVID-19. That said, the pandemic has continued beyond the initial wave(s), with varying lockdown restrictions extending into 2022 (see New Zealand Government, 2021), particularly with the emergence of more transmissible variants of the virus (World Health Organization, 2022). Moreover, our results provide general insight into the effects of government restrictions on citizens’ relative perceptions of inequality. As such, the current study provides a springboard for future research to investigate the relationship between the pandemic and relative deprivation well beyond the initial COVID-19 outbreak.

## Conclusion

The present study examines the effects of the COVID-19 pandemic on IRD and GRD and whether these effects were disproportionately felt by the structurally disadvantaged. Our results reveal that, relative to the pre-pandemic control group, the strictest Alert Levels increased IRD among the general population but selectively increased GRD among minorities. Moreover, the pandemic was indirectly associated with status-specific support for collective action and income redistribution via IRD and GRD. These results demonstrate that salient forms of inequality elicit individual- and group-based comparisons and suggest that the pandemic presents unique opportunities for social change. The current study thus provides the foundations for future research examining the consequences of the pandemic on relative deprivation and associated individual- and group-based responses to inequality.

## Supplemental Material

sj-docx-1-spp-10.1177_19485506231163016 – Supplemental material for Status-Based Asymmetries in Relative Deprivation During the COVID-19 Pandemic Click here for additional data file. Supplemental material, sj-docx-1-spp-10.1177_19485506231163016 for Status-Based Asymmetries in Relative Deprivation During the COVID-19 Pandemic by Kieren J. Lilly, Chris G. Sibley and Danny Osborne in Social Psychological and Personality Science

## Notes

1. While Auckland returned to Alert Level 3, the remainder of the country returned to Alert Level 2. Businesses outside of Auckland remained open and experienced less disruption than in the stricter Alert Levels.

2. As the pandemic has made objective economic conditions more salient, we also examined the moderating effects of socioeconomic status (SES). Although SES was negatively associated with both IRD (*b* = −0.220, *SE* = 0.006, *p* < .001) and GRD (*b* = −0.067, *SE* = 0.006, *p* < .001), no interactions between SES and Alert Levels were significant (*p*s ≥ .086).

3. Given the disproportionate impact of the pandemic on women, we also examined the moderating effects of gender on the association between the different Alert Levels and IRD. Similar to the results for ethnicity, IRD did not differ across women and men at any Alert Level (*p*s ≥ .152).
