# Supplementary material for: Status-Based Asymmetries in Relative Deprivation During the COVID-19 Pandemic
Source: Soc Psychol Personal Sci. 2023 Apr 4:19485506231163016. doi: 10.1177/19485506231163016 (PMC10076160; doi:10.1177/19485506231163016)
Supplement: sj-docx-1-spp-10.1177_19485506231163016 – Supplemental material for Status-Based Asymmetries in Relative Deprivation During the COVID-19 Pandemic [file sj-docx-1-spp-10.1177_19485506231163016.docx]

**Supplementary Materials**

Table of Contents

[Table S1 2](#_Toc126573226)

[Propensity Score Matching Variables 2](#_Toc126573227)

[Table S2 3](#_Toc126573228)

[Multiple Regression Analyses Predicting Support for Collective Action During the COVID-19 Pandemic 3](#_Toc126573229)

[Table S3 4](#_Toc126573230)

[Multiple Regression Analyses Predicting Support for Income Redistribution During the COVID-19 Pandemic 4](#_Toc126573231)

[Table S4 5](#_Toc126573232)

[Indirect Effects of the COVID-19 Pandemic on Support for Collective Action via Individual-based Relative Deprivation (IRD) and Group-based Relative Deprivation (GRD) 5](#_Toc126573233)

[Table S5 6](#_Toc126573234)

[Indirect Effects of the COVID-19 Pandemic on Support for Income Redistribution via Individual-based Relative Deprivation (IRD) and Group-based Relative Deprivation (GRD) 6](#_Toc126573235)

[References 7](#_Toc126573236)

# Table S1

## Propensity Score Matching Variables

| **Variable** | **Coding** | **Item(s)** | **Reference** |
| --- | --- | --- | --- |
| Gender | 0 (women), 1 (men), 0.5 (gender diverse) | “What is your gender?” (open-ended) | Fraser et al. (2020) |
| Birth year | Year of birth | “What is your date of birth?” (open-ended) |  |
| Ethnicity |  | Coded from “Which ethnic group(s) do you belong to?” (Open-ended and tick boxes) | Statistics New Zealand (2020) |
| European | 0 (no), 1 (yes) |  |  |
| Māori | 0 (no), 1 (yes) |  |  |
| Pacific | 0 (no), 1 (yes) |  |  |
| Asian | 0 (no), 1 (yes) |  |  |
| Born in NZ | 0 (no), 1 (yes) | Coded from “Which country were you born in?” (open-ended) |  |
| NZ Citizen | 0 (no), 1 (yes) | “Are you a New Zealand Citizen?” (yes/no) |  |
| Education (NZREG) | 0 (low), 10 (high) | Coded from “What is your highest level of qualification?” (open-ended) | New Zealand Qualifications Authority (2012) |
| New Zealand Deprivation Index (NZDep) | 0 (low), 10 (high) | Coded based on participant address | Atkinson et al. (2014) |
| New Zealand Socioeconomic Index (NZSEI) | 0 (low), 90 (high) | Coded based on participant education, income, and occupation | Fahy et al. (2017) |
| Urban | 0 (no), 1 (yes) | Coded based on participant address |  |
| Religious | 0 (no), 1 (yes) | “Do you identify with a religious and/or spiritual group?” (yes/no) | Hoverd and Sibley (2010) |
| Partner | 0 (no), 1 (yes) | Coded from “What is your relationship status?” (open-ended) |  |
| Parent | 0 (no), 1 (yes) | Coded from “How many children have you given birth to, fathered, or adopted?” (open-ended) |  |
| Smoker | 0 (no), 1 (yes) | “Do you currently…smoke tobacco cigarettes?” (yes/no) |  |
| Disability | 0 (no), 1 (yes) | “Do you have a health condition or disability that limits you, and that has lasted for 6+ months?” (yes/no) |  |
| Diagnosis |  | “Have you been diagnosed with, or treated for, any of the following health conditions by a doctor in the last five years?” | Lee et al. (2017) |
| Depression | 0 (no), 1 (yes) | “Depression” (tick box) |  |
| Anxiety | 0 (no), 1 (yes) | “Anxiety disorder” (tick box) |  |

# Table S2

## Regression Analyses Predicting Support for Collective Action During the COVID-19 Pandemic

|  | **Collective Action Support** | | | | | | | |
| --- | --- | --- | --- | --- | --- | --- | --- | --- |
|  | **Ethnic Majority** | | | | **Ethnic Minority** | | | |
|  | *B* | SE | *p* | β | *B* | SE | *p* | β |
|  |  |  |  |  |  |  |  |  |
| Intercept | 1.121^***^ | 0.017 | < .001 | 0.982^***^ | 1.136^***^ | 0.069 | < .001 | 0.617^***^ |
| Alert Level 4 | 0.018 | 0.024 | .439 | 0.006 | 0.109 | 0.070 | .121 | 0.020 |
| Alert Level 3 | 0.029 | 0.034 | .382 | 0.006 | 0.073 | 0.109 | .504 | 0.009 |
| Alert Level 2 | 0.034 | 0.031 | .273 | 0.008 | 0.143 | 0.099 | .148 | 0.019 |
| Alert Level 1 | 0.054^*^ | 0.022 | .015 | 0.017^*^ | 0.003 | 0.061 | .962 | 0.001 |
| Alert Level 3^1^ | –0.006 | 0.032 | .844 | –0.001 | 0.091 | 0.098 | .354 | 0.012 |
| IRD | 0.051^***^ | 0.005 | < .001 | 0.069^***^ | –0.010 | 0.016 | .546 | –0.008 |
| GRD | 0.332^***^ | 0.009 | < .001 | 0.314^***^ | 0.619^***^ | 0.013 | < .001 | 0.617^***^ |
|  |  |  |  |  |  |  |  |  |

*Note.* ^1^Alert Level 3 in Auckland only, Alert Level 2 elsewhere. ^*^*p* ≤ .05, ^**^*p* ≤ .01, ^***^*p* ≤ .001.

# Table S3

|  | **Support for Income Redistribution** | | | | | | | |
| --- | --- | --- | --- | --- | --- | --- | --- | --- |
|  | **Ethnic Majority** | | | | **Ethnic Minority** | | | |
|  | *B* | SE | *p* | β | *B* | SE | *p* | β |
|  |  |  |  |  |  |  |  |  |
| Intercept | 3.994^***^ | 0.041 | < .001 | 2.049^***^ | 3.193^***^ | 0.097 | < .001 | 1.681^***^ |
| Alert Level 4 | 0.324^***^ | 0.040 | < .001 | 0.058^***^ | 0.325^***^ | 0.089 | < .001 | 0.058^**^ |
| Alert Level 3 | 0.493^***^ | 0.058 | < .001 | 0.062^***^ | 0.350^**^ | 0.133 | .009 | 0.043^*^ |
| Alert Level 2 | 0.444^***^ | 0.054 | < .001 | 0.060^***^ | 0.194 | 0.126 | .122 | 0.025 |
| Alert Level 1 | –0.216^***^ | 0.040 | < .001 | –0.040^***^ | –0.099 | 0.079 | .207 | –0.021 |
| Alert Level 3^1^ | 0.225^***^ | 0.061 | < .001 | 0.028^***^ | 0.242 | 0.133 | .069 | 0.030 |
| IRD | 0.135^***^ | 0.009 | < .001 | 0.107^***^ | 0.148^***^ | 0.020 | < .001 | 0.124^***^ |
| GRD | –0.053^***^ | 0.013 | < .001 | –0.029^***^ | 0.216^***^ | 0.017 | < .001 | 0.208^***^ |
|  |  |  |  |  |  |  |  |  |

## Regression Analyses Predicting Support for Income Redistribution During the COVID-19 Pandemic

*Note.* ^1^Alert Level 3 in Auckland only, Alert Level 2 elsewhere. ^*^p < .05, ^**^p < .01, ^***^p < .001.

# Table S4

## Indirect Effects of the COVID-19 Pandemic on Support for Collective Action via Individual-based Relative Deprivation (IRD) and Group-based Relative Deprivation (GRD)

|  | **Collective Action Support** | | | | | | | |  |
| --- | --- | --- | --- | --- | --- | --- | --- | --- | --- |
|  | **Ethnic Majority** | | | | **Ethnic Minority** | | | | |
|  | *Indirect effect* | SE | 95% CI | *p* | *Indirect effect* | SE | 95% CI | *p* | |
| AL4 → IRD | **0.006^***^** | 0.002 | [0.003, 0.010] | .001 | 0.001 | 0.002 | [–0.002, 0.008] | .625 | |
| AL3 → IRD | **0.007^**^** | 0.003 | [0.003, 0.012] | .004 | –0.001 | 0.003 | [–0.010, 0.002] | .740 | |
| AL2 → IRD | **0.009^***^** | 0.002 | [0.005, 0.014] | < .001 | 0.001 | 0.002 | [–0.002, 0.009] | .732 | |
| AL1 → IRD | –0.001 | 0.002 | [–0.004, 0.002] | .475 | 0.000 | 0.001 | [–0.002, 0.004] | .929 | |
| AL3^1^ → IRD | 0.002 | 0.002 | [–0.002, 0.007] | .403 | –0.001 | 0.003 | [–0.010, 0.002] | .773 | |
|  |  |  |  |  |  |  |  |  | |
| AL4 → GRD | 0.008 | 0.007 | [–0.006, 0.023] | .266 | 0.092 | 0.056 | [–0.022, 0.199] | .103 | |
| AL3 → GRD | 0.009 | 0.011 | [–0.012, 0.029] | .428 | 0.136 | 0.081 | [–0.020, 0.299] | .093 | |
| AL2 → GRD | 0.003 | 0.010 | [–0.016, 0.024] | .740 | **0.173^**^** | 0.082 | [0.015, 0.338] | .034 | |
| AL1 → GRD | **0.023^**^** | 0.007 | [0.008, 0.037] | .002 | **0.114^**^** | 0.047 | [0.024, 0.207] | .015 | |
| AL3^1^ → GRD | 0.010 | 0.011 | [–0.010, 0.031] | .354 | **0.258^**^** | 0.084 | [0.087, 0.417] | .002 | |

*Note.* ^1^Alert Level 3 in Auckland only, Alert Level 2 elsewhere. 95% CI = 95% Confidence Intervals. Indirect effects were estimated using 5000 bootstrapped resamples (with replacement). Values highlighted in bold reflect significant indirect effects.

^*^*p* < .05, ^**^*p* < .01, ^***^*p* < .001.

# Table S5

## Indirect Effects of the COVID-19 Pandemic on Support for Income Redistribution via Individual-based Relative Deprivation (IRD) and Group-based Relative Deprivation (GRD)

|  | **Support for Income Redistribution** | | | | | | | |
| --- | --- | --- | --- | --- | --- | --- | --- | --- |
|  | **Ethnic Majority** | | | | **Ethnic Minority** | | | |
|  | *Indirect effect* | SE | 95% CI | *p* | *Indirect effect* | SE | 95% CI | *p* |
| AL4 → IRD | **0.016^***^** | 0.005 | [0.007, 0.025] | < .001 | –0.017 | 0.012 | [–0.042, 0.004] | .137 |
| AL3 → IRD | **0.019^**^** | 0.006 | [0.007, 0.032] | .003 | 0.013 | 0.017 | [–0.021, 0.048] | .456 |
| AL2 → IRD | **0.024^***^** | 0.006 | [0.012, 0.036] | < .001 | –0.013 | 0.016 | [–0.046, 0.017] | .415 |
| AL1 → IRD | –0.003 | 0.004 | [–0.012, 0.005] | .471 | –0.002 | 0.010 | [–0.022, 0.018] | .854 |
| AL3^1^ → IRD | 0.005 | 0.006 | [–0.007, 0.018] | .404 | 0.011 | 0.018 | [–0.023, 0.047] | .519 |
|  |  |  |  |  |  |  |  |  |
| AL4 → GRD | –0.001 | 0.001 | [–0.005, 0.001] | .311 | 0.031 | 0.020 | [–0.006, 0.070] | .112 |
| AL3 → GRD | –0.001 | 0.002 | [–0.006, 0.002] | .461 | 0.049 | 0.029 | [–0.005, 0.109] | .088 |
| AL2 → GRD | –0.001 | 0.002 | [–0.004, 0.003] | .762 | **0.062^*^** | 0.029 | [0.006, 0.121] | .033 |
| AL1 → GRD | **–0.004^*^** | 0.002 | [–0.007, –0.001] | .017 | **0.040^*^** | 0.017 | [0.009, 0.073] | .015 |
| AL3^1^ → GRD | –0.002 | 0.002 | [–0.006, 0.001] | .379 | **0.090^**^** | 0.030 | [0.034, 0.153] | .003 |

*Note.* ^1^Alert Level 3 in Auckland only, Alert Level 2 elsewhere. 95% CI = 95% Confidence Intervals. Indirect effects were estimated using 5000 bootstrapped resamples (with replacement). Values highlighted in bold reflect significant indirect effects.

^*^*p* < .05, ^**^*p* < .01, ^***^*p* < .001.

# References

Atkinson, J., Salmond, C., & Crampton, P. (2014). *NZDep2013 index of deprivation*. Department of Public Health, University of Otago.

Fahy, K., Lee, A., & Milne, B. (2017). *New Zealand socio-economic index 2013* (0908350813). <http://www.stats.govt.nz/methods/research-papers/nz-socio-economic-index-2013.aspx>

Fraser, G., Bulbulia, J., Greaves, L. M., Wilson, M. S., & Sibley, C. G. (2020). Coding Responses to an Open-ended Gender Measure in a New Zealand National Sample. *The Journal of Sex Research*, *57*(8), 979-986. <https://doi.org/10.1080/00224499.2019.1687640>

Hoverd, W. J., & Sibley, C. G. (2010). Religious and Denominational Diversity in New Zealand 2009. *New Zealand Sociology*, *25*(2), 59-87. <https://search.informit.org/doi/10.3316/informit.113861382782746>

Lee, C. H., Duck, I. M., & Sibley, C. G. (2017). Ethnic inequality in diagnosis with depression and anxiety disorders. *The New Zealand Medical Journal*, *130*(1454), 10-20.

New Zealand Qualifications Authority. (2012). *The New Zealand qualifications framework*. Wellington, New Zealand: New Zealand Government Retrieved from <https://www.nzqa.govt.nz/assets/Studying-in-NZ/New-Zealand-Qualification-Framework/requirements-nzqf.pdf>

Statistics New Zealand. (2020). *Ethnicity.* <http://archive.stats.govt.nz/methods/classifications-and-standards/classification-related-stats-standards/ethnicity.aspx>
